# The Law Dealing with the Erection of Small-Pox Hospitals
*An address delivered by Mr. Alexander Macmorran, K.C., before the Royal Institute of Public Health on Thursday, July 13.


**Published:** 1905-07-22

**Authors:** 


### THE LAW DEALING WITH THE ERECTION OF SMALL-POX HOSPITALS.*

Mr. Macmorran pointed out the difficulties connected with the erection of these hospitals. Wherever such a hospital is erected the value of the neighbouring property goes down, falling sometimes to one-half of its former value. But yet it is quite lawful to erect such a hospital, and the person aggrieved has no remedy unless he can show that it is a nuisance. If he cannot prove that it is a nuisance, his only course is when the local authority applies to the Local Government Board for the money, to come forward and object, and the Local Government Board can refuse to sanction the building of the hospital.

The leading case on this subject was Managers of the Metropolitan Asylum District *v.* Hill (6 A.C. 19), which was tried before Baron Pollock, and subsequently went up to the House of Lords. The action was brought by certain owners of property at Hampstead against the managers of the Metropolitan Asylum, for having erected a small-pox hospital near their properties, which was a nuisance, and had carried on the said hospital so as to be a nuisance. Baron Pollock held, first, that the Metropolitan Poor Act, 1867, under which they had erected the hospital, did not make the defendants mere irresponsible agents to carry out the orders of the Local Government Board so as to exempt them from liability. Secondly, that they were not exempt on the ground that they acted *bonâ fide* in the discharge of a duty cast on them by the Act. The House of Lords supported this view, holding that the District Board could not set up the statutes nor the orders of the Poor Law Board under it, as an answer to an action or to prevent an injunction issuing to restrain the Board from continuing the nuisance. Lord Blackburn said:—" On those who seek to establish that the Legislature intended to take away the private rights of individuals lies the burden of showing that such an intention appears by express words or necessary implication. In Fleet *v.* Metropolitan Asylums District (1 *T. L. R.* 80), the defendants established within 685 yards of the plaintiff's house a camp for small-pox patients. The plaintiff alleged that the health of the neighbourhood had been endangered by the camp, and that the camp was a nuisance, and claimed an injunction to restrain the defendants from maintaining the same. It was held that the plaintiff had failed to prove that there was any danger to him or those residing upon his property, and that the action must be dismissed. Another case was that of Saunders *v.* Mayor of New Windsor (*L. T. News*, Sept. 18, 1886, p. 353), which was an action brought to restrain the use of certain lands as a small-pox hospital, so as to cause a nuisance. Mr. Justice Stirling, before whom the case was tried, said that the law in respect of this was clear, and was laid down so long ago as Lord Hardwicke in Baines *v.* Baker. In that case "the plaintiff was owner of building land in the neighbourhood, and gave evidence (of something which seems very probable) that the fears of infection from the proposed hospital greatly deteriorated the letting value of his property. Lord Hardwicke refused to grant an injunction, saying, what is undoubtedly law, that loss arising from the fears of mankind, though in themselves reasonable, would not create a nuisance at law, and that before he could grant an injunction he must be satisfied that what was proposed to be done would be a legal nuisance affecting the plaintiff's private rights." Now Mr. Justice Stirling, in the course of his judgment in Saunders *v.* Mayor of Windsor, said that there was evidence in the case of the fears of mankind, but he could not on that account grant an injunction. It was necessary for that purpose to establish the existence of a legal nuisance. What did the evidence of the plaintiff amount to? "His observations had shown that the incidence of small-pox on persons living at a distance from a quarter to half a mile of a small-pox hospital was nearly three and a half times the incidence of small-pox upon those livin at a distance of more than one mile from such hospital, and even the latter incidence was greater than it would be had there been no small-pox hospital within this distance. Then, although by careful administration it might be possible to prevent the extension of small-pox from the hospital of the defendant authorities, arising from personal communication with infected persons or infected articles within the hospital, there was every reason to regard persons resident within one mile of the hospital as exposed to greater risk of infection by small-pox, when persons suffering from the disease were aggregated within the said hospital. Further, the plaintiff's family, living within a distance of from a quarter to half a mile of a hospital of the nature of the defendant authori-

* An address delivered by Mr. Alexander Macmorran, K.C., before the Royal Institute of Public Health on Thursday, July 13.

ties' hospital, containing even a small number of persons suffering from small-pox in the acute stage of the disease, was, under the circumstances, exposed to risks of infection, either from infection aerially conveyed or from personal communication with other persons who, as the result of exposure to such infection, should come to suffer from small-pox." On that evidence Mr. Justice Stirling said he was asked to grant an injunction, but his lordship thought that the fears of mankind would be best allayed by his refusing to grant an injunction.

There are great differences of opinion amongst medical experts as to the danger of these small-pox hospitals. Some experts say that there is danger within a radius of $2\frac{1}{2}$ miles, others within half a mile, and others only a few feet. The judges say that so long as there are these wide differences of opinion amongst experts, it is impossible for them to say whether a hospital is a danger to the district, and therefore unless it can be proved to be a legal nuisance, they will not interfere.

